# What Lies Beneath? The Role of Community Engagement in Translating COVID-19 Research Findings to Policy-Makers

**DOI:** 10.34172/ijhpm.2024.8249

**Published:** 2024-04-20

**Authors:** Bronwen Merner, Sophie Hill, Freya Saich, Ariane Virgona, Defeng Jin, Alisa Pedrana, Coral Keren, Rachel Kar Yee Chung, Deborah Osborne, Anna Lee Wilkinson, Alison Coelho, Lisa Gibbs, Katherine B. Gibney, Margaret Hellard, Dean Lusher, Rebecca Ryan

**Affiliations:** ^1^Centre for Health Communication and Participation, School of Psychology and Public Health, La Trobe University, Melbourne, VIC, Australia.; ^2^Burnet Institute, Melbourne, VIC, Australia.; ^3^Disease Elimination, Burnet Institute, Melbourne, VIC, Australia.; ^4^School of Public Health and Preventive Medicine, Monash University, Melbourne, VIC, Australia.; ^5^Community Engagement Group, Optimise Study, Burnet Institute, Melbourne, VIC, Australia.; ^6^Melbourne School of Population and Global Health, University of Melbourne, Melbourne, VIC, Australia.; ^7^Coelho Networks, Melbourne, VIC, Australia.; ^8^Centre for Disaster Management and Public Safety, University of Melbourne, Melbourne, VIC, Australia.; ^9^Victorian Infectious Diseases Service, Royal Melbourne Hospital, and Department of Infectious Diseases, University of Melbourne, at the Peter Doherty Institute for Infection and Immunity, Victoria, Australia.; ^10^Department of Infectious Diseases, University of Melbourne, Peter Doherty Institute for Infection and Immunity, Melbourne, VIC, Australia.; ^11^Department of Infectious Diseases, The Alfred and Monash University, Melbourne, VIC, Australia.; ^12^Doherty Institute and Melbourne School of Population and Global Health, University of Melbourne, Melbourne, VIC, Australia.; ^13^Centre for Transformative Innovation, Swinburne University of Technology, Melbourne, VIC, Australia.

**Keywords:** Community Participation, Public Health, Health Policy, Implementation Science, COVID-19, Australia

## Abstract

**Background:** Community engagement is key to developing local and context-specific strategies for the prevention and control of COVID-19. However, expedited research design and approval in the early days of the pandemic may have limited the opportunities for community members to influence pandemic-related research. In this study, we sought to understand how a Community Engagement Group (CEG) could impact a large longitudinal COVID-19 research project (Optimise), when involved solely in the interpretation and knowledge translation phases of the research.

**Methods:** Seven community members were recruited for the CEG, representing a diverse range of groups. Each month, Optimise data of topical importance were compiled into a draft report. The CEG discussed the draft report at their monthly meeting and members’ contributions were incorporated into the final report for distribution to policy-makers. In this study, a document analysis was undertaken of ten consecutive reports produced between February and November 2021. Each report was compared pre- and post- the inclusion of CEG contributions, which were then analysed using thematic analysis.

**Results:** Community engagement in the interpretation and knowledge translation phases of Optimise had positive impacts on reports for policy-makers, including grounding the empirical findings in broader community perspectives, identifying policy issues affecting different groups and contributing unique insights beyond the empirical findings. Overall, the CEG contributions demonstrated the complexity of lived experience lying beneath the empirical data.

**Conclusion:** Community engagement in the translation of the Optimise findings resulted in research reports to policy-makers that were reflective of a broader range of community perspectives, and that provided potential solutions to emerging policy issues related to COVID-19. This study adds to the evidence base about the impact of community engagement in the later interpretation and knowledge translation phases of research, particularly in the context of reporting to policy-makers during a public health emergency.

## Background

**Key Messages**

**Implications for policy makers**

- In public health emergencies, community engagement at the interpretation and knowledge translation phases of a research project can positively impact the research.
- The establishment of a community engagement group (CEG) that meets regularly within a pandemic-related research project can be a useful and practical way of gaining insights into the policy implications of the research findings from different sectors of the community.
- Community members are ideally placed to identify policy issues and gaps, including inequities, and generate creative solutions.
- Policy approaches to COVID-19 need to be tailored to specific groups within communities to avoid inadvertently disadvantaging particular groups or making it difficult to adhere with public health measures.

**Implications for the public**

Community engagement is key to developing appropriate strategies for preventing and controlling the spread of COVID-19. However, during the early days of the pandemic, research could be designed and approved rapidly, so there was often less opportunity for the community to be involved in shaping the research. In this article, we explored if, and how, a Community Engagement Group (CEG) could impact COVID-19 research (the Optimise Study) when they were involved solely at the later stages of the research process. The results showed the CEG contributed to the interpretation and translation of the findings by grounding the research in broader community perspectives, using the findings to identify policy issues and create solutions, and contributing unique insights beyond the research findings. This study builds on evidence demonstrating that community members’ lived experiences can complement the results of empirical research, making the findings more meaningful to real world settings.

The COVID-19 pandemic has seen policy-makers experience unprecedented pressure to make rapid decisions for their populations, often in a context of uncertainty and with a risk of long-term consequences.^1^ To make meaningful decisions, they require timely evidence which explicitly considers the relevance of the findings to the local context.^2^ Researchers are typically most skilled at placing their research findings within academic literature, but generally less adept at understanding and articulating how the findings relate to the broader community and political context.^3^ Engaging community members throughout the research process can help to address this gap.^4^

 Previous research has shown community engagement is key to developing local and context-specific strategies for the prevention and control of COVID-19.^5^ In a commentary early in the pandemic, Marston et al reported that community members were aware of misinformation and rumours circulating in their communities, as well as stigma and structural barriers to protective measure uptake. They also have practical experience of the difficulties caused by government restrictions and are ideally placed to develop collective responses.^6^ Community engagement in research is also important for building public trust in scientific evidence.^7^ During COVID-19, fostering public trust in public health authorities and the information provided has been particularly important for facilitating adherence to public health strategies.^8^

 In this article, we describe the community engagement approach used during the Optimise Study, a longitudinal study of COVID-19 in Victoria, Australia, and report on analysis of the impacts of this engagement on the monthly study reports prepared for policy-makers. The Optimising Isolation, Quarantine and Distancing (Optimise) Study for COVID-19 was a research platform exploring how Victorians experienced COVID-19 and responded to government measures. The study also monitored the unintended consequences of public health measures for preventing or reducing the spread of the virus. Commencing in September 2020 and concluding in December 2022, the project was led by the Burnet and Doherty Institutes in Melbourne, Australia.^9^

 The Optimise Study established a Community Engagement Group (CEG), consisting of seven community members, to review the monthly findings of the study, and make recommendations to policy-makers to tailor public health messages to priority groups. Although standards of community engagement in research emphasize that community members should be involved from the earliest stage of the research process, the Optimise Study was designed rapidly, early in the COVID-19 pandemic, before the community engagement approach was decided.^10^ Thus, by the time the CEG was formed, opportunities to be involved in the earlier stages of the research (such as deciding the study objectives and methods) had passed.

 Expedited research design and approval processes, as experienced in the Optimise Study, were common in the early days of COVID-19, which may have limited the capacity for early engagement in pandemic-related research more broadly.^11,12^ Given the potential for future pandemics, the Optimise CEG approach provides an opportunity to explore how community engagement purely in the later stages of a project, can influence the research (if at all).

 Existing research has shown positive impacts can be achieved in the later stages of research, when the community has also been involved in the earlier stages of the project. In the data analysis and interpretation phases, positive impacts of engagement have included an increase in the relevance of the results to community needs, a greater awareness of social context in interpreting the results, and the identification of research gaps.^13-15^ In the dissemination stage, impacts have included the provision of wider, more culturally relevant viewpoints in study reports, and increased credibility of reports among stakeholders.^13^ However, it is unclear whether similar impacts would be evident in projects where engagement occurred purely in the later stages, such as the Optimise Study.

 In this article, we explore how the CEG’s engagement in the interpretation and knowledge translation phases of the Optimise Study impacted the research reports prepared for policy-makers. The findings of this research contribute to the evidence base about the impact of community engagement on the research process during public health emergencies when expedited research design and approval processes may limit earlier opportunities for involvement.^5,16^

###  The Optimise Study and the Establishment of the Community Engagement Group 

 To understand the context of the CEG’s establishment and role, we begin by outlining the three main components of the Optimise Study.^17,18^ The first component was a longitudinal cohort study that explored the experiences of approximately 1000 Victorian adults, focusing on people with recent COVID-19 infection (or their close contacts), those at higher risk of COVID-19 infection and/or the unintended consequences of public health measures to reduce transmission. For the duration of the study, each participant completed a monthly quantitative questionnaire and weekly diary that included their experiences of COVID-19 and their understanding of and adherence to public health measures.

 Secondly, smaller groups of participants were purposively selected from the longitudinal cohort to participate in in-depth interviews at key time points across the pandemic.

 The third component, and the subject of this article, was the formation of the CEG. The CEG was established to facilitate the involvement of community members in the Optimise Study. The group’s role was to contribute to the monthly-reporting cycle of Optimise (see below) by reviewing the findings and making recommendations to policy-makers to tailor public health messages to priority groups.

 CEG members were recruited through advertisements emailed to community organisations as well as professional networks. A purposive sampling frame was used to select members who belonged to the following priority groups targeted by the Optimise Study: people with lived experience of COVID-19, healthcare workers, culturally and linguistically diverse groups, international students, people from regional centres, people with chronic illness, youth (18 to 24 years) and older people (aged 60 years and over). Attempts to recruit members of two further priority groups (aged care and factory workers) were unsuccessful.

 Seven CEG members were recruited. Although each member belonged to a priority population, they also brought their own rich biographical history and interests to the group.^19^ The group consisted of four men and three women, across a range of ages (from 19 to over 60 years of age). Three of the members were parents or grandparents and two were from culturally and linguistically diverse backgrounds (Greek and Malaysian). One member identified as LGBTIQA+ (Lesbian, Gay, Bisexual, Transgender, Intersex, Queer, and Asexual, with the plus symbol representing other sexual orientations not explicitly included in the initialism). Four members were employed (full-time, part-time or casually), two were students (one secondary school student and one postgraduate student) and one member had retired from paid work. Members lived in a range of accommodation types, including private, government and emergency housing.

 Consistent with best practice, the participation of CEG members was facilitated in a range of ways.^20^ Firstly, members were asked to nominate their preferred meeting times and days each month and the research team aimed to meet the preferences of most participants. This resulted in meetings being held on different days and times each month to maximize participation. Those who could not attend the meeting in person were encouraged to submit written feedback in advance, which was incorporated into the CEG discussion. All meetings were held virtually which allowed representation from across the state of Victoria. CEG members also received a stipend of $AUD 115 per meeting in recognition of their expertize and time (including pre-reading of the draft report). On average, five CEG members attended the monthly Zoom meeting with an additional two members contributing written comments.

###  CEG Involvement in the Rapid Reporting Cycle

 Due to the rapidly changing nature of the pandemic, the Optimise Study adopted a rapid analysis and reporting cycle to facilitate timely use of emerging data to inform policy-makers and decision making related to the COVID-19 response. The CEG met monthly as part of the report cycle (shown in Figure).

**Figure:**
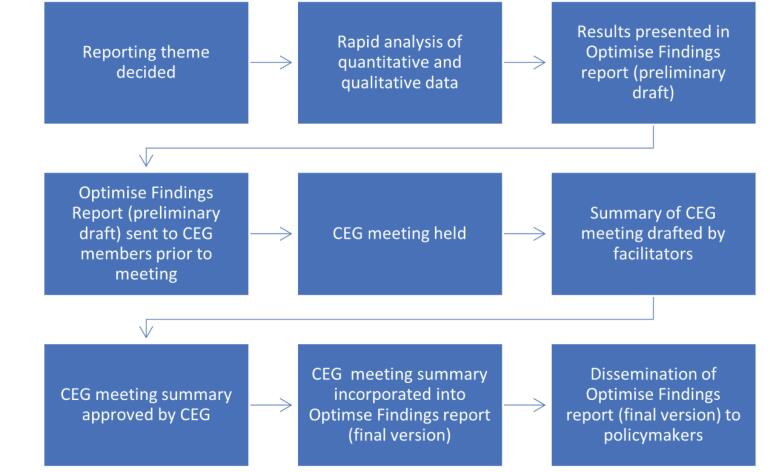
Monthly Report Cycle for Optimise Study. Abbreviation: CEG, Community Engagement Group.

 A reporting theme was decided by the Optimise Executive Group each month, to reflect a topic of critical importance to the community and/or policy-makers at that time. The Optimise Executive Group included one of the CEG facilitators (a senior researcher specialized in community engagement) who contributed the CEG’s perspectives on relevant topics each month. A range of topics were chosen over the course of the Optimise Study including testing patterns and behaviours, information and communication needs, perceptions of infection likelihood and vaccination uptake (See Table 1).

**Table 1:** Monthly Report Topic and Community Engagement Group Discussion Questions

**Report No.**	**Report Topic**^a^	**Month & Year**	**CEG Discussion Questions**
3	COVID-19 testing and strategies to improve testing uptake	Feb 21	1. What are your general reflections about the report from the perspective of your community? 2. One of the key findings of this report is that people are reporting symptoms but not getting tested. Do you feel this is a problem in your community? If so, why do you feel people are reluctant to get tested? 3. In your community, how can we encourage more people with symptoms to get tested?
4	Vaccine preparedness	Mar 21	1. From the perspective of your community, what are your reflections about any aspect of the Optimise Study’s findings on vaccine preparedness? 2. Are there issues or topics raised by the group in response to the first question, that you'd like to discuss further?
5	Social network and mixing patterns	Apr 21	1. What are your reflections about any aspect of the Optimise Study’s findings on social networks and mixing patterns? 2. If you are getting out and about more now, what has helped to increase your confidence about doing so? Amongst your networks, which people are still being careful about mixing and why? Is there anything that government or non-government stakeholders can do to assist people to feel more confident about mixing? 3. The government is increasingly encouraging people to return to their pre-COVID activities (eg, going back to the workplace, visiting the city centre, using public transport, plane travel). How comfortable and confident do you feel about returning to these activities? Are you starting to make plans into the future or are you focusing on shorter term achievements?
6	Gender and mental health	May 21	1. What are your reflections about any aspect of the Optimise Study’s findings on gender? 2. Are you having any conversations about COVID-19 related mental health concerns and ways of coping in your communities? Are the issues raised different between genders? Or do they vary according to other characteristics such as employment, income, social support etc? Are there groups who are particularly at-risk of mental health issues related to COVID-19 in your communities? 3. At the height of the pandemic, there was more emphasis in government and non-government messaging about mental health and coping. Now the focus is on people returning to their pre-COVID-19 activities, such as going back to work and tourism. At this stage of the pandemic, what messages (if any) should the government and community organisations be conveying about mental health and coping strategies? And to whom should these messages be targeted?
7	Income and finances	Jun 21	1. What are your reflections about any aspect of the Optimise Study’s findings on income and finances? 2. How have members of your community been impacted by changes to income and/or finances? 3. How secure do members of your community feel their income and finances are? What would help to increase feelings of security?
8	Impact of government restrictions on risk mitigation strategies	Jul 21	1. Social distancing: Do you still think about physical distancing in your daily interactions? Does this change during lockdown? When do you find it most difficult to maintain social distancing? What strategies or cues help you to maintain social distancing (eg, signage, markers on the floor of shops, other)? 2. Testing: The report shows only one in three people who had COVID-19 symptoms in June got tested. What is your current decision-making process for getting a test? Has this changed over the course of the pandemic? Does being in lockdown affect your likelihood of getting a test? What are the current barriers to getting tested? How could testing be made easier? 3. Lockdown strategies: What helps you most to get through lockdowns? Is the current lockdown easier or harder than previous lockdowns? What would help you to get through future lockdowns?
9	Vaccination knowledge, attitudes and beliefs	Aug 21	1. Vaccine information and communication: Has the availability of reliable information about the vaccine changed since earlier this year? What strategies have been most effective in communicating the information? What strategies have not worked? 2. Motivating factors to get the vaccine: If you are not eligible to be vaccinated yet, what factors would nudge you and others in your community to get the vaccination once you are eligible? If you have already been vaccinated, what factors would nudge other eligible people in your community to get the vaccine? 3. Mandatory vaccination: Mandatory vaccination is currently being considered by government and private employers in Australia (eg, aged care facilities, SPC Ardmona in Shepparton). If mandatory vaccinations are implemented (across Victoria, or in a workplace, for example), what might be the issues from your community’s perspective?
10	Information access and communication (plus discussion of vaccine uptake strategies)	Sep 21	1. Information access and communication: What do you think are the main implications of the findings on information access for your community? What could government or community leaders do to address these implications (if anything)? 2. Vaccine uptake: With the recent spread of the delta strain of COVID-19 in Victoria, the government is rapidly trying to increase the number of Victorians who are vaccinated. A number of strategies have been suggested to improve vaccine uptake in people who may be reluctant or haven’t got onto it yet. What do you think of each of the following strategies, and would they help to encourage vaccination among reluctant people in your community? Do you think people in your community who have already been vaccinated would have a problem with the above strategies being introduced now? eg, would they be resentful they were not paid to have the vaccine etc? If any of the above strategies were introduced, what would be the best ways to communicate they were available in your community? eg, via social media, press conference etc?
11	Testing	Oct 21	1. What do you understand are the benefits of getting tested now many in the community are vaccinated? You can mention the benefits to yourself, your family/friends and the broader population. 2. What do you want to know about rapid testing? 3. In the context of Victoria opening up again, what are the key issues for your community? How could these be addressed?
12	COVID-19 testing in schools and attitudes and concerns about the current state of the pandemic	Nov 21	1. Now that Victoria has been out of lockdown for a few weeks, what issues are arising for your community?

Abbreviation: CEG, Community Engagement Group. ^a^All reports are publicly available at https://optimisecovid.com.au/study-findings/.

 Each month, quantitative data from the longitudinal cohort study relevant to the theme were rapidly analysed, together with qualitative data from the in-depth interviews where these coincided. Data was then synthesized and presented in the Optimise Findings report (preliminary draft). An example of a preliminary draft report is shown in Supplementary file 1. The draft was similar in structure to the final version (shown in Supplementary file 2), with findings presented in narrative text, diagrams and tables. However, due to the tight timelines of the Optimise reporting cycles, in a small number of cases, the draft did not include all data sources that were relevant, but these were included at a later point and formed part of the final report. The Recommendations section was also incomplete at this stage.

 The CEG received the preliminary draft, and discussion questions, via email one week prior to its scheduled monthly meeting. The discussion questions were formulated collaboratively by Optimise researchers who were involved in the data collection and analysis process, and the CEG facilitators, who were researchers specialized in community engagement. The questions were based on the content of the preliminary draft report and designed to achieve a collaborative level of involvement from the CEG, according to the IAP2 Spectrum of Public Participation.^21^ This meant the questions prompted the CEG to provide advice and generate solutions to problems raised in the findings, which were then incorporated into the Optimise Study final report to the maximum extent possible. A list of the topics and the discussion questions for each CEG meeting are shown in Table 1.

 Each CEG meeting was held via Zoom for 90 minutes. In early meetings, CEG members were invited to respond to discussion questions by the meeting facilitator. As the group developed familiarity over the course of the monthly meetings, they also began asking each other questions and responding to the comments of other CEG representatives. As well as the two meeting facilitators, two Optimise Study researchers were present in the meetings to answer questions about the study and COVID-19 more generally, and to ask follow-up questions of CEG members. All meetings were audio-recorded so a comprehensive summary of the discussion (CEG meeting summary) could be drafted by the facilitators. Some direct quotations were transcribed verbatim and included in the summary. An example of a CEG meeting summary is shown in Supplementary file 3. Once drafted, the summary was emailed to all CEG members for feedback.

 Following incorporation of feedback from CEG members, content from the CEG meeting summary was integrated into the Optimise Findings report (final version) by Optimise researchers. Generally, the parts of the summary that pertained to specific findings in the Optimise report were added to the relevant sections of the report. When the CEG raised issues that were not already included as part of the report findings, these were added under a separate heading. CEG recommendations were also added to the Recommendations section at the end of the report. Supplementary file 4 shows an example of how the CEG contributions were integrated into a final report (CEG contributions are highlighted in yellow). Once complete, the final version of the Optimise Findings report was then emailed to policy-makers directly, as well as published and made publicly available on the Optimise webpage.^22^

## Methods

 We aimed to understand the CEG’s contributions to the Optimise Study’s monthly findings reports to policy-makers. To do this, we compared the preliminary and final Optimise Study monthly reports, conducting an analysis of documents relating to the monthly CEG meetings and Optimise Study monthly findings report over a 10-month period (February to November 2021). We used the READ approach to document analysis which consists of the following four stages: (1) ready the materials; (2) data extraction; (3) data analysis; and (4) distil the findings.^23^ Each of these stages are described below.

 Stage 1: Ready the materials: This stage involved determining the parameters for the nature and number of documents to be included in analysis. As the research focused on the role of the CEG in the monthly reporting cycle, we determined that all documents contributing to the CEG meeting, as well as the Optimise monthly findings report, would be included. These documents are outlined in Table 2.

**Table 2:** Data Sources Used for Document Analysis

**Name of Report**	**Description **	**Purpose**	**Circulation**
Optimise monthly report: preliminary draft	The first draft of the Optimise monthly report. The draft included data from the Optimise monthly survey and individual interviews/diary entries when available	Used to formulate CEG discussion questions. Reviewed by CEG members one week prior to the CEG meeting.	CEG members
CEG discussion questions	Questions determined by the CEG facilitators and Optimise researchers prior to the CEG meeting	Helped CEG participants prepare for the discussion. Reviewed by CEG members one week prior to the CEG meeting.	CEG members
CEG monthly meeting report	Summary of the CEG meeting discussion	Informed the final Optimise monthly report. Excerpts used in the final monthly report.	CEG members, Optimise Executive Group
Optimise monthly report: final version	Combination of preliminary draft plus insights from CEG monthly meeting report	Informed policy-makers about findings of Optimise project and perspectives of the CEG on the findings.	Policy-makers across the Victorian and Commonwealth Governments and other stakeholders, Optimise Study website

Abbreviation: CEG, Community Engagement Group.

 As identified data sources were interdependent and focused on the same reporting theme, we determined the unit of analysis would be the four documents contributing to the reporting theme, rather than the individual document source.^24^ Thus, for the reporting theme of “vaccination,” the unit of analysis included the Optimise findings report (preliminary draft), CEG discussion questions, CEG meeting summary and Optimise findings report (final version) on the topic of vaccination.

 We purposively sampled ten consecutive reporting themes, which led to the inclusion of 40 documents in the analysis. This sample was chosen because it reflected the continuation of a consistent CEG cohort over a period of ten months.

 Step 2: Data extraction: For each reporting theme, the associated data sources were read superficially in chronological order (from preliminary draft to final version) to understand the context. At this stage, we extracted the following data into an Excel spreadsheet: month, reporting theme, discussion questions. CEG contributions to the final report were identified by searching the text for “Community Engagement Group” or “CEG.” Once identified, the CEG contributions to the report (and surrounding text) were extracted for thematic analysis.^25^

 Step 3: Data analysis: BM, who was a facilitator of the CEG, used thematic analysis to code how each CEG contribution was used as part of the broader report.^25^ Initial codes included “formulating report recommendations,” “explaining quantitative data,” “providing a contemporary view on topic.” Similar codes were then compared and contrasted, and grouped into themes. After ten reporting cycles had been analyzed, data saturation had been achieved and data collection and analysis was ceased. SH, who was not involved with the CEG, reviewed the initial analysis and identified some duplication between themes. As a result, BM refined the themes, which were then re-checked by SH.

 Step 4: Distil the findings: When BM and SH had reached consensus that the themes were robust, the findings were sent to the remaining co-authors (including two CEG members) for their feedback. The themes were finalized once this feedback had been incorporated.

## Results

 The results of the document analysis demonstrated the CEG contributed to the monthly Optimise report of findings by grounding the empirical findings in broader community perspectives, identifying policy issues and generating potential solutions, and contributing unique insights beyond the empirical findings. Each of these contributions is elaborated below and supported by excerpts from the final version of the monthly reports.

###  Grounding the Empirical Findings in Broader Community Perspectives 

 Through contextualising, validating and supplementing the empirical findings, the CEG grounded the empirical data in their lived experiences. The CEG contextualized the empirical findings by highlighting the different social meanings associated with COVID-19 and the government’s response. Uncovering these social meanings conveyed the complexity of managing the pandemic. For example, the CEG conveyed the high level of stigma associated with COVID-19 in the community in the early stages of the pandemic by adding the following insight into Report 3:

*“The shame and stigma regarding having COVID-19 is pervasive throughout the community. Participants identified a range of settings where they or people they know have experienced discrimination or shame for having COVID-19. Participants noted that the media often sensationalize stories of people with COVID-19 and that people with the disease are ‘bombarded with hate.’ Online and via social media, people use hashtags such as #covidiot to publicly shame and harass others who have supposedly ‘done wrong’”* (Report 3, February 2021).

 Further to this, CEG members reported how the experience of stigma shaped people’s responses to seeking testing:

*“For young people at school, if there is a ‘COVID-scare,’ there is intense focus on identifying the person who had COVID but [people] are less interested in knowing whether that person is ok. Young people are scared to get a test or be diagnosed with COVID-19 for fear that ‘people will come after them’ and they do not want to be the one person who gets COVID-19. In some culturally and linguistically diverse communities, family groups and communities can blame and stigmatise individuals who try to get tested by asking ‘What have you been doing [to get COVID19]? Who are your friends?’; which may encourage them not to seek out testing in the future” *(Report 3, February 2021).

 The CEG also reported how groups could perceive changes in government restrictions differently. In the following excerpt, the representative of healthcare workers emphasised how her community perceived the end of lockdowns compared to others:

*“The representative for healthcare workers reported that whilst others in the community are getting back to normal, healthcare workers are still directly impacted by COVID-19 every day. She described that many healthcare workers had mixed emotions about coming out of lockdown. They felt positive about seeing family and friends but were also apprehensive about having less restrictions: ‘I feel like I’m walking through this minefield of trying to live this normal life that we’re all so keen to get back to, but on the other side knowing it could all go pear-shaped again from a work point of view’” *(Report 12, November 2021).

 As well as providing context, the CEG also helped to validate the empirical findings of the Optimise Study. For example, after a long COVID lockdown during the Delta strain and the achievement (for a brief period) of COVID-zero, the CEG agreed with the empirical finding that relaxation of mask-wearing had increased people’s confidence to go out:

*“This may explain some of the recent increases in [social] contacts, with the removal of the requirement to wear masks in most settings other than public transport. This was reiterated in the Community Engagement Group meeting… where the loosening of mask wearing restrictions was reported as having increased people’s confidence to get back out into the community” *(Report 5, June 2021).

 Sometimes, a CEG member or members disagreed with the empirical findings. For example, in responding to the empirical finding that the top three sources of pandemic information were the daily government press conference (70%), news media (68%) and health authorities (60%), the representative for young people said this was not reflective of his community which “used social media rather than news or government sites for information. He felt teachers needed to be given up-to-date information to pass onto students” (report 10, September 2021). In this way, the CEG enriched the empirical data by highlighting the experiences of specific communities that differed from the majority.

 Perspectives of the CEG also supplemented the empirical data, particularly the qualitative interviews, demonstrating the data could apply to the community more broadly:

*“Participants who undertook qualitative interviews, and members of the Community Engagement Group described a wide range of causes contributing to poor mental health for both themselves and amongst their respective communities”* (Report 6, July 2021).

###  Identifying Policy Issues and Generating Potential Solutions 

 When discussing monthly topics, the CEG also demonstrated that members could identify issues with the government’s pandemic response. Issues included a lack of communication about policies, as well as unintended social consequences. Sometimes, these issues related to the community as a whole, whereas others were restricted to specific groups. For example, when discussing the empirical data about mental health during the pandemic, the participants identified the inadequacy of mental health services:

*“Participants of the Community Engagement Group described the challenges they and people in their networks had encountered when seeking mental health services, including difficulties finding an available psychologist, knowing where to seek appropriate help and information, and accessing the appropriate care during an acute mental health crisis. The representative for healthcare workers reported that some [healthcare workers] were coping with the impacts of COVID-19 by reducing their hours, changing jobs, or changing the area in which they work in order to cope with the ongoing stress of COVID-19”* (Report 6, July 2021).

 In another example, when discussing findings about the information sources people used for COVID-19, participants felt the Victorian Government’s COVID-19 website was difficult to navigate and thus they sought alternative sources of information:

*“A couple [of participants] preferred to use websites [other than the official government website] that distilled the information [about exposure sites] in a more accessible way. One example cited was COVID-19 Near Me - Victoria ( covid19nearme.com.au ) and exposure site bots on Twitter” *(Report 10, September 2021).

 Other gaps related to inequities experienced by specific groups represented within the CEG. In a meeting that focused on the financial impacts of COVID-19, the representative of international students reported the significant hardship facing her community:

*“…many international students were experiencing financial hardship because they had lost financial support from their families, and their work hours were restricted due to student visa requirements. Foodbanks and universities provided much-needed assistance for them. Other temporary visa holders were also at greater risk as they had no access to government benefits”* (Report 7, June 2021).

 The CEG also demonstrated how existing social inequities could potentially affect the willingness of certain communities to respond to government advice about the pandemic. For example, the representative of people in crisis accommodation reported many in his community did not trust the government and were unlikely to seek COVID-19 information from government websites. His community also perceived they would be the “last in line” to be offered the first COVID-19 vaccination (Report 4, March 2021).

 As well as pointing out issues or gaps, the CEG also suggested practical solutions. Some solutions were relevant to the community generally. For example, following a finding in the empirical data that adherence to social distancing was greater in regional than metropolitan areas, the group suggested that COVID-19 marshals could be employed in busy parts of the city (such as train stations and supermarkets) to politely remind people to adhere to social distancing (Report 8, July 2021). They also suggested ways to improve the community’s adherence to stay-at-home rules:

*“…it would be helpful to provide more information from ‘behind-the-scenes’ eg*,* how many people contact tracers need to contact because of one party. This could be presented using visual infographics to emphasise the gravity of just one case in the community”* (Report 9, August 2021).

 In response to concerns in the community about the vaccine, the CEG provided a range of recommendations for improving uptake:

*“While information is available on the Department of Health website, participants suggested that information about the vaccines and the rollout strategy should be disseminated via the channels people use to access information about COVID-19 and recognised a role for the COVID-Safe app. Participants also stated that some groups such as people who are homeless or people from culturally and linguistically diverse backgrounds, may not trust the government and will require such information to come from other sources they consider credible. In such cases, information from a trusted GP [general practitioner] or a community leader would be more reassuring than information disseminated via the media or listed on government websites” *(Report 4, March 2021).

 The CEG was able to compare and contrast the government’s response across different time points of the pandemic. This included acknowledging when the response had improved:

*“Community Engagement Participants noted some recent improvements to vaccine information, including the greater availability of easier to understand information. More written information and information sessions were also reported as being available in community languages. Participants noted more targeted vaccination advertising was occurring on social media as well as for specific groups ( eg, temporary or student visa holders)” *(Report 9, August 2021).

###  Contributing Unique Community Insights Beyond the Empirical Findings 

 The CEG also constituted a unique data source about the pandemic beyond the empirical findings. Sometimes, the group was asked to provide their views on government policy approaches prior to their introduction (eg, rapid testing in schools, vaccination mandates). These topics had not been assessed by either the quantitative or qualitative parts of the empirical research. For these topics, the CEG provided a valuable mechanism for obtaining a unique cross-section of community views on the likely feasibility and acceptability of such measures. For example, regarding vaccination mandates, the representative for young people in crisis accommodation felt there was likely to be some resistance which would not change “unless the government proves they are trustworthy and looking out for us underdogs” (Report 9, August 2021). In contrast, the representative for older people reported her community was more likely to respond positively to the mandates, given older people were more at risk of dying from the virus, and had also witnessed previous pandemics (eg, polio) (report 9, August 2021).

 In a further example, prior to the introduction of rapid tests, the CEG identified concerns that were already emerging in the community:

*“The participant representing people with chronic illness stated that rapid testing was another way the government was putting the onus for COVID management onto individuals, similar to home quarantine. He expressed concern that people would not comply to the same extent” *(Report 11, October 2021).

## Discussion

 Our results demonstrated that involving community members purely in the interpretation and knowledge translation phases of a study can have positive impacts on the research summaries communicated to policy-makers. The findings showed that the CEG impacted the Optimise Study reports in three key ways. The group grounded the empirical findings in broader community perspectives, used the findings to identify policy issues and solutions, and also contributed unique insights beyond the empirical findings. In each of these ways, the community members added value by demonstrating the rich complexity of lived experience that lay beneath and beyond the empirical results.^19^ These results are similar to previously reported impacts of community engagement in later research stages, when engagement has also occurred earlier in the research. For example, previous studies have found that community engagement in the interpretation and dissemination phases has helped to contextualise the results, increase their relevance to community needs and identified research gaps.^13-15^ However, our study extends the previous literature by demonstrating that positive impacts can also be achieved when the community is involved purely at the later stages. Although engagement throughout the research process is commensurate with best practice, the results suggest the community can still contribute meaningfully to research when public health emergencies, such as COVID-19, eliminate earlier opportunities for involvement.

 The CEG’s contributions aligned with policy-makers’ known preferences for receiving research evidence, including that the applicability of the findings to the local context was considered, and the complexity underlying the findings was communicated.^2,3^ The CEG also bridged the gap between the community and researchers by streamlining the process for identifying the community implications of the research findings.^26^ Previous research demonstrates that policy-makers prefer research summaries that include explicit attention to implementation and equity considerations.^2^ As COVID-19 had an unequal impact on minority groups, the CEG provided an opportunity for members from priority (including minority) groups to ensure key recommendations from research were relevant and therefore more likely to be impactful. The group’s insights showed that policy approaches needed to be tailored to ensure specific communities were not inadvertently disadvantaged or unable to adhere to public health guidance.^8^ The CEG also highlighted numerous issues in the implementation of public health guidance, including a lack of communication and limited access to support services.^8^ The group was also able to formulate practical, community-based solutions to inform policy-makers’ responses. This finding is consistent with previous research that demonstrated community members engaged in thinking “outside of the box” and applied a fresh perspective to problem-solving.^26^

 There are specific characteristics of the CEG model that may have facilitated or limited its influence on the Optimise Study. Firstly, the group met monthly for ten consecutive months which promoted both cohesion and flexibility within the group. Over time, members could adapt easily to consider the new content presented at each meeting. The group also became more knowledgeable about COVID-19 policies over time, so they were able to suggest solutions that were more likely to be useful to policy-makers. The CEG was diverse, including members from different genders, across a range of ages, in different employment and housing circumstances and with a rich array of additional life experiences. This diversity allowed the CEG to provide a snapshot view of the impacts of COVID-19 policies on different people at a single point in time. Such varied responses to government COVID-19 policies are supported by previous literature.^27^ However, as CEG membership focused on the priority populations set by the Optimise Study, not all potentially marginalised groups within the community were included (eg, people with disabilities, people with limited English proficiency or Aboriginal and Torres Strait Islander people). The small group size also limited the range of views that could be contributed about each priority population by the CEG members.

###  Areas for Further Research

 The findings of this study would have been strengthened by a formal evaluation of policy-makers’ use of the Optimise Study findings reports. However, this was beyond the scope of the Optimise Study and the CEG study as one component. Future research might investigate how the contributions of community members to research reports influence policy-makers’ perceptions and use of research findings. Another topic for future research is how the CEG’s contributions shaped the translation of the Optimise Study results to different groups eg, did CEG participation increase the members’ understanding of COVID-19 and associated public health guidance, and did any increase in knowledge filter through to their community group? Additionally, exploring how CEG involvement affected both CEG members and researchers over time would also be beneficial.

## Conclusion

 In fast-paced policy and research environments, such as during a pandemic, community engagement in research can be neglected. However, the results of this study show that involving community members in COVID-19 research at the knowledge translation phase is practically possible and can have positive impacts on the research. The results of the study showed that community members contributed to the research reports to policy-makers by grounding the empirical findings in broader community perspectives, identifying policy issues and generating potential solutions, and contributing unique insights beyond the empirical findings. Each of these contributions added value by demonstrating the complexity of lived experience that lay beneath and beyond the empirical results. This research adds to the evidence base demonstrating the impact of community involvement in the knowledge translation phase of research – a less studied, but equally important, component of community engagement in research.

## Acknowledgements

 We would like to thank all members of the Optimise Study Community Engagement Group. Optimise is a partnership between the Burnet Institute and Peter Doherty Institute in collaboration with The University of Melbourne, Swinburne University of Technology, La Trobe University, Monash University, Victorian Department of Health and Human Services, Centre for Culture Ethnicity and Health, Health Issues Centre, and Royal Children’s Hospital, Independent Multicultural Consultant. The authors gratefully acknowledge the generosity of the community members who participated in the study. The authors appreciatively acknowledge the work of all Optimise project team members and collaborators who have contributed to the ongoing delivery of the study.

## Ethical issues

 The involvement of the CEG in the Optimise Study received ethical approval from La Trobe University Human Research Ethics Committee (approval no. HEC20532). The broader Optimise Study received ethical approval from The Alfred Human Research Ethics Committee (approval no. 333/20).

## Competing interests

 Authors declare that they have no competing interests.

## Funding

 Optimise received funding support from the Victorian Government Department of Jobs, Precincts and Regions, the Victorian Department of Health, the Macquarie Group Foundation, and Burnet Institute donors. The authors gratefully acknowledge the contribution to this work of the Victorian Operational Infrastructure Support Program received by the Burnet Institute. MH and KBG receive funding support from National Health and Medical Research Council Investigator grants. AC was contracted to the Optimise Study to provide expert guidance about diversity and inclusion in recruitment procedures and study design.

## Supplementary files


Supplementary file 1. Example of Preliminary Draft Report.

Supplementary file 2. Example of Final Report.

Supplementary file 3. Example of CEG Meeting Summary.

Supplementary file 4. Example of CEG Contributions Integrated Into Final Report.
