# Supplementary material for: What Lies Beneath? The Role of Community Engagement in Translating COVID-19 Research Findings to Policy-Makers
Source: Int J Health Policy Manag. 2024 Apr 20;13:8249. doi: 10.34172/ijhpm.2024.8249 (PMC11270602; doi:10.34172/ijhpm.2024.8249)
Supplement: Supplementary file 1 — Example of Preliminary Draft Report. [file ijhpm-13-8249-s001.pdf]

The Optimise Study: Income and finances

DRAFT

Report 7 | June 2021

Optimise

Burnet Institute
Medical Research. Practical Action.

Doherty Institute

REPORT 7 | JUNE 2021

The Optimise Study is a partnership between Burnet Institute and Doherty Institute in collaboration with University of Melbourne, Swinburne University of Technology, Monash University, La Trobe University, Murdoch Children's Research Institute, the Centre for Ethnicity and Health, and the Health Issues Centre.

Optimise is a longitudinal cohort study that will follow up to 1000 participants for a 12-month period. Study participants are not intended to be representative of the broader population but instead have been intentionally recruited from key groups who are considered to be:

- at risk of contracting COVID-19
- at risk of developing severe COVID-19 or,
- at risk of the unintended consequences of the restrictions (see page 6 for a comprehensive list of these key groups).

Participants are then asked to nominate people who play a key role in their lives, and where permission is given, these people are also invited to participate in the study. Establishing a map of social connections is important because it can be used to examine the influence of the social network on an individual or key groups 1) behaviour including adhering to government directions on COVID-19, 2) attitudes and level of engagement in key COVID-19 interventions such as testing and vaccination, and 3) experience of the unintended consequences of COVID itself, or the government restrictions imposed due to COVID-19. The resulting social map increases our understanding of the interplay between the individual, social and community-level impacts of COVID-19. For more detail on the Optimise study please visit https://optimisecovid.com.au/

Gendered differences in coping and responding to COVID-19
This report explores the impact of COVID-19 on income and finances.

542
SURVEY PARTICIPANTS

16
SEMI-STRUCTURED INTERVIEWS

1
COMMUNITY ENGAGEMENT GROUP MEETING

This report draws on the findings from a number of Optimise research activities. These include:

- responses from 500 participants who completed the Optimise baseline survey, follow up surveys and contact diaries between 14 September 2020 and 1 June 2021
- phone-based semi-structured qualitative interviews conducted with a subset of survey participants (N=9) in December 2020 (n=7) and May 2021 (n=9)
- a Community Engagement Group meeting facilitated by the Centre for Health Communication and Participation at La Trobe University on 15 June 2021.

OPTIMISE COHORT

SUMMARY AND IMPLICATIONS

COVID-19 has had a significant impact on income and finances for many people in our study. In May, the majority of our cohort were employed in some capacity or had retired, while 8% were unemployed. Young people were most likely to be unemployed than any other age group. Many participants have had a change of income or work since the start of the pandemic. Furthermore, one in three participants in our study reported experiencing some form of financial hardship since March 2020 with at least 40% requiring financial support from family or friends. Despite these stressors, there has also been 'silver linings' with some participants reporting improvements in their work-life balance while others, such as essential workers, expressed gratitude at their ongoing ability to work and earn an income.

EMPLOYMENT STATUS

In May, 33% of participants reported being employed full-time, 32% worked part-time while 5% were self-employed and 18% were retired. Eight percent were unemployed with young people aged 18-25 the most likely to be unemployed in our study. In May 22% of young people were unemployed however this increased from 13% in April.

Employment status of participants by age

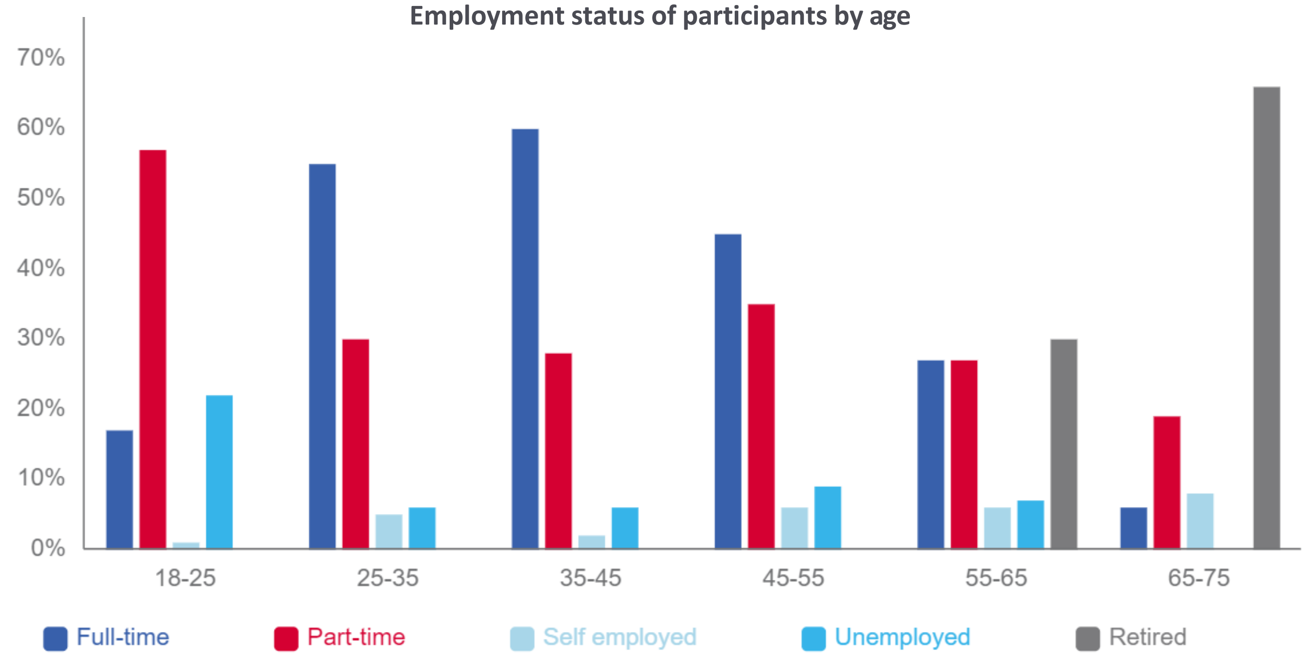

CHANGES TO WORK

Two thirds of participants had experienced some change to their work since the start of the pandemic with the majority experiencing a change in hours (83%) or change income (85%). Approximately 41% had a job loss while just over half had a new job.

Overall, COVID-19 had an adverse impact on income and finances for many of the qualitative participants. One participant spoke about how COVID-19 job losses exacerbated the distress caused by the 2019/2020 Black Summer bushfires. For another participant who had to move from full-time to part-time work, she

> *"… so that we were really heightened in that our family's in danger, your work's in danger… we started the year in such a high adrenaline fuelled fashion and then it didn't really improve. So, I think that probably made the whole year even harder, just because you're in that fight or flight state for such a long period of time. So yeah, we were already on the edge well and truly before this."*

> "So, I went down to part time from full time work since March last year… yes monetarily, we've been hit hard, because I'm earning way less but there's the benefits of that too where I got to, you know, relax and sort of live a little bit more instead of working. I really did enjoy… having time to go for walks, having time for me to catch up… in doing things around the house that you never have time to do because you work full time … "

noted that while this caused a financial impact from her loss of income, there were also some positives in terms of her work-life balance.

Despite the many adverse impacts on finance and income, some participants felt their income had not been affected and that their life mostly carried on as normal for them, especially those who were essential workers. Many of these people expressed gratitude for their circumstances during the pandemic and conveyed a theme of positivity on reflection of their COVID-19 experience.

ENTERING THE FIELD AND CAREER CHANGES

> "I was doing a marketing job prior to COVID and then I kind of came to the realization that it wasn't an essential job and not very stable. So, then that's why I'm doing a Master's…but it was actually for the better because marketing was never a real passion."

For some qualitative interview participants, the impacts of COVID-19 on job security and income made some people reassess their career choice. One participant described how the pandemic made it difficult to find a job in marketing and that this prompted her to reflect on her career direction more broadly.

> "…it was very hard to even just get interviews…I think no one was leaving their jobs, so there was no new jobs. … And then the ones that were a job that we had essentially 2 years of graduates trying to get them instead of one year, so it was this huge build-up of people just trying to get like one job."

Another participant was made redundant due to COVID-19 when working in the travel industry with job security a key motivating factor in changing his career. Similarly, another participant who also worked in the travel industry spoke about the employment uncertainty COVID-19 created when it came to returning to work after maternity level and hoping "there's still a job when I'm done."

Several participants spoke about the challenges of completing placements for degrees or finding a job especially for entry-level positions.

FINANCIAL HARDSHIP

One in three participants in our study reported experiencing some form of financial hardship since March 2020. At least 40% of participants required financial support from family and friends, while 31% were unable to pay their utility bill and 31% received assistance from welfare or community organisations. Over time, there has been a downward trend of participants requiring financial help from friends or family however, the proportion of people unable to pay their utilities bill increased between November and April.

Financial hardship of participants over time

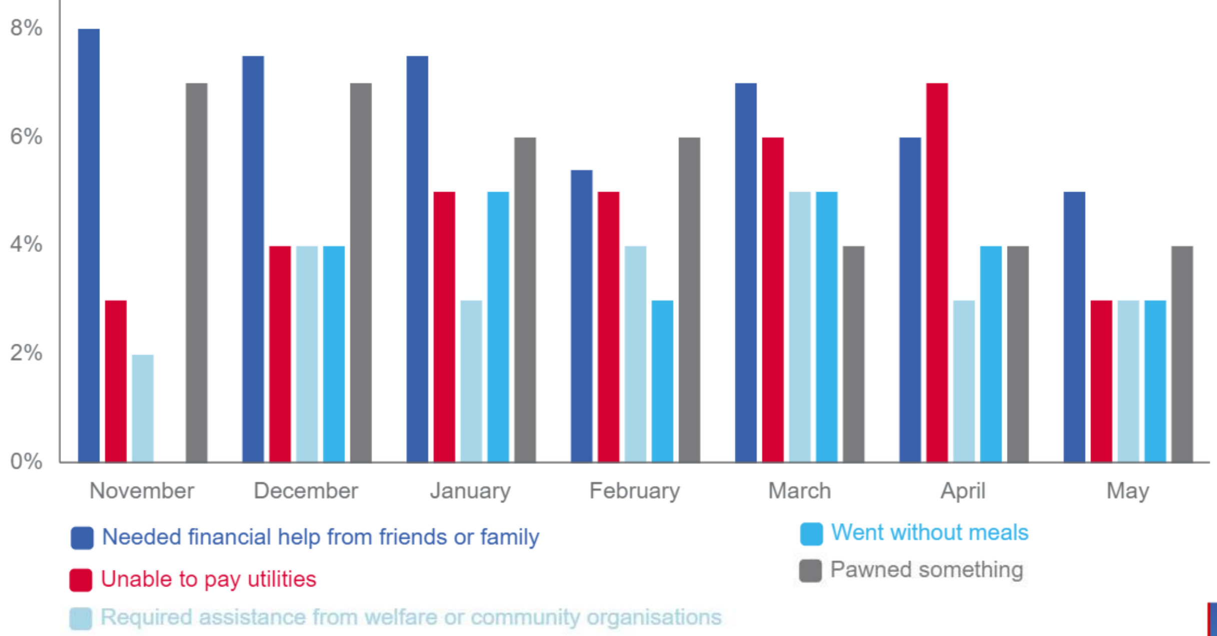

Some qualitative interview participants spoke about the support they provided to others, received from others, or received from the government during times of financial hardship in the pandemic. Participants raised the importance of support from family and friends both financially and as a support system.

"if I didn't have my parents throughout the pandemic I don't know what I would do…"

FINANCIAL SUPPORT

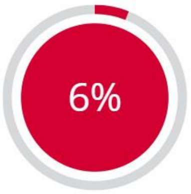

6% of participants reported accessing financial support.

When asked whether they had accessed financial support, 6% of participants reported they had. Women were slightly more likely to report accessing financial support than men (7% and 3% respectively). A higher proportion of young people aged 18-25 accessed financial support than any other age group.

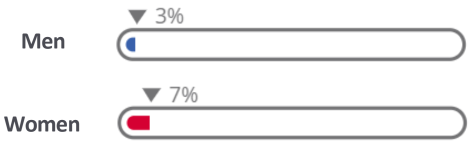

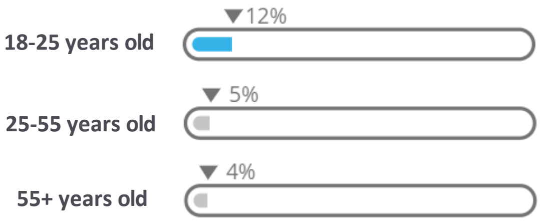

The qualitative interview participants noted the importance of government income support payments after losing work. However, one participant spoke about the uncertainty of government financial support at the start of the pandemic and ongoing concerns that financial support would be removed or reduced with limited warning. In addition, another mentioned the need to persevere when applying for income support payments after her daughter was initially deemed ineligible.

"It kind of felt like every press conference was just kind of waiting to see, 'Oh, are they going to drop the rate? Are they going to remove the eligibility?' …it felt like you were very much beholden to someone else's idea of how much support someone who's lost their job because of coronavirus would be…" - University student

RECOMMENDATIONS

REPORT PREPARED BY

Ms Freya Saich
Mr Edwin Kwong
Dr Katherine Heath
Ms Aimée Altermatt
Dr Stephanie Munari
Dr Bronwen Merner
Assoc Professor Sophie Hill
Dr Alisa Pedrana
Dr Katherine Gibney
Professor Margaret Hellard

ACKNOWLEDGEMENTS

Participants of the Community Engagement Group
Participants of the Qualitative Interviews
Optimise Data Collectors
Optimise Data Management Team
Optimise Qualitative Working Group
Optimise Knowledge Translation and Policy Working Group
Optimise Executive Committee

Burnet Institute
85 Commercial Road
Melbourne, Australia, 3004

burnet.edu.au

The Peter Doherty Institute
for Infection and Immunity
792 Elizabeth Street
Melbourne, Australia, 3000

doherty.edu.au

Chief Investigators

Professor Margaret Hellard AM
margaret.hellard@burnet.edu.au
+61 3 9282 2111

Dr Katherine Gibney
katherine.gibney@unimelb.edu.au
(03) 9035 3958

For More Information

Stephanie Fletcher-Lartey
Study Coordinator
stephanie.fletcher@burnet.edu.au
