# Supplementary material for: What Lies Beneath? The Role of Community Engagement in Translating COVID-19 Research Findings to Policy-Makers
Source: Int J Health Policy Manag. 2024 Apr 20;13:8249. doi: 10.34172/ijhpm.2024.8249 (PMC11270602; doi:10.34172/ijhpm.2024.8249)
Supplement: Supplementary file 2 — Example of Final Report. [file ijhpm-13-8249-s002.pdf]

The Optimise Study: The impact of COVID-19 on income and finances

Report 7 | June 2021

REPORT 7 | JUNE 2021

The Optimise Study is a partnership between Burnet Institute and Doherty Institute in collaboration with University of Melbourne, Swinburne University of Technology, Monash University, La Trobe University, Murdoch Children's Research Institute, the Centre for Culture Ethnicity and Health, and the Health Issues Centre.

Optimise is a longitudinal cohort study that will follow up to 1000 participants for a 12-month period. Study participants are not intended to be representative of the broader population but instead have been intentionally recruited from key groups who are considered to be:

- at risk of contracting COVID-19,
- at risk of developing severe COVID-19, or
- at risk of the unintended consequences of the restrictions

Participants are then asked to nominate people who play a key role in their lives, and where permission is given, these people are also invited to participate in the study. Establishing a map of social connections is important because it can be used to examine the influence of the social network on an individual or key groups 1) behaviour including adhering to government directions on COVID-19, 2) attitudes and level of engagement in key COVID-19 interventions such as testing and vaccination, and 3) experience of the unintended consequences of COVID itself, or the government restrictions imposed due to COVID-19. The resulting social map increases our understanding of the interplay between the individual, social and community-level impacts of COVID-19.
For more detail on the Optimise study please visit https://optimisecovid.com.au/

The impact of COVID-19 on income and finances

This report explores the impact of COVID-19 on income and finances in terms of:

- Employment status
- Changes to work
- Entering the field and career changes
- Financial hardship and access to financial support

542	16	1
SURVEY PARTICIPANTS	SEMI-STRUCTURED INTERVIEWS	COMMUNITY ENGAGEMENT GROUP MEETING

This report draws on the findings from a number of Optimise research activities. These include:

- responses from 542 participants who completed the Optimise baseline survey, follow up surveys and contact diaries between 14 September 2020 and 1 June 2021
- phone-based semi-structured qualitative interviews (N=16) conducted with a subset of survey participants conducted in December 2020 (n=7) and May 2021 (n=9)
- a Community Engagement Group meeting facilitated by the Centre for Health Communication and Participation at La Trobe University on 15 June 2021.

For the first time we have also included findings from the COVID-19 Work and Health Study – a national cohort of 2603 working-age Australians during 2020 and into 2021, conducted by researchers at Monash University School of Public Health and Preventative Medicine.

OPTIMISE COHORT

CHANGES TO WORK DURING THE COVID-19 PANDEMIC

Two thirds of participants completing the quantitative surveys reported some change to their work since the start of the pandemic with the majority experiencing a change in hours (83%) or change in income (85%). Approximately 41% had experienced a job loss, however, by May 2021, 66% of those participants had later found work, 2% had retired and 7% reported being unemployed. Of the participants who had found new work since the pandemic 24% of those new jobs were in healthcare, 12% were in education, while 11% were in accommodation/hospitality and 10% were in retail.

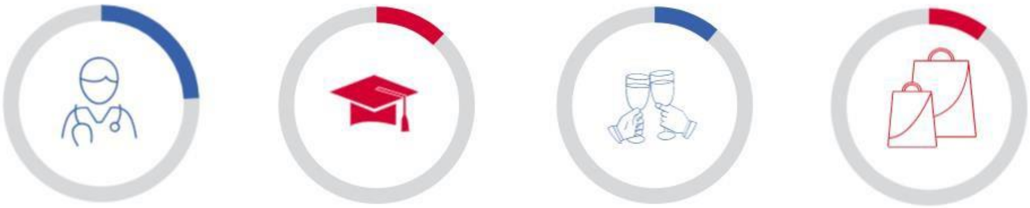

Healthcare (24%) **Education (12%)** **Hospitality (11%)** **Retail (10%)**

In May 2021, 33% of participants reported being employed full-time, 32% worked part-time, 5% were self-employed and 18% were retired. Eight percent were unemployed, with young people aged 18-24 the most likely to be unemployed in our study. In May 2021, 22% of young people were unemployed increasing from 13% in April 2021, likely a consequence of the most recent circuit breaker lockdown in Victoria (late May).

Employment status of participants by age in May 2021

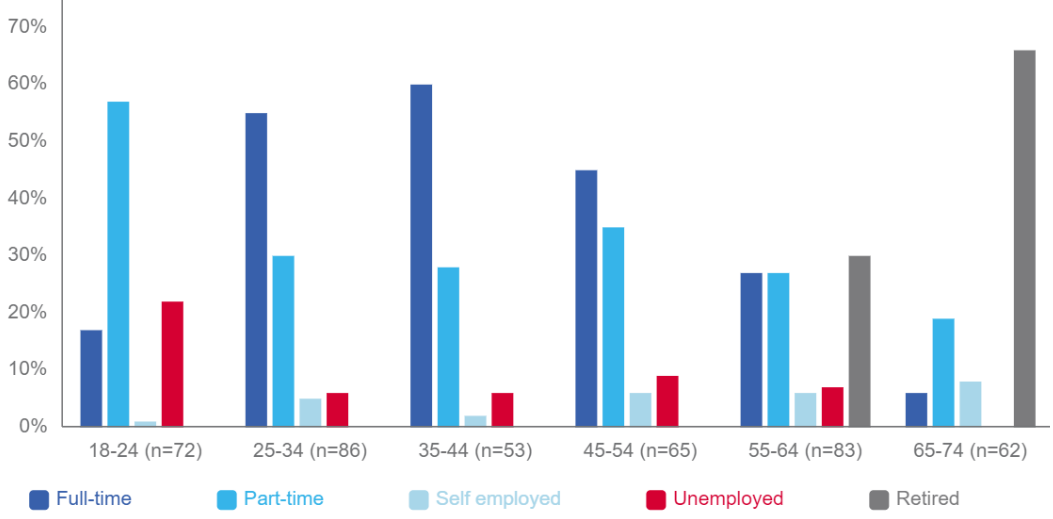

"So, I went down to part time from full time work since March last year… yes monetarily, we've been hit hard, because I'm earning way less but there's the benefits of that too where I got to, you know, relax and sort of live a little bit more instead of working… "

Many of the participants who completed a qualitative interview reported COVID-19 had adversely impacted their income and finances. One participant who had to move from full-time to part-time work noted that while this caused a financial impact from her loss of income, there were also some positives in terms of her work-life balance.

Another participant who was impacted by the 2019/2020 Black Summer bushfires spoke about how COVID-19 job losses exacerbated the distress and anxiety caused by the bushfires. Other participants, particularly essential workers whose income had not been affected, reported that their life mostly carried on as normal for them. Upon reflection, many of these participants expressed gratitude for their financial circumstances and job

"… so that we were really heightened in that our family's in danger, your work's in danger… So, I think that probably made the whole year even harder, just because you're in that fight or flight state for such a long period of time."

security during the pandemic and conveyed a sense of appreciation for their situation compared to others, throughout the COVID-19 pandemic.

CHALLENGES ENTERING THE JOB MARKET AND FORCED CAREER CHANGES

> *"I was doing a marketing job prior to COVID and then I kind of came to the realisation that it wasn't an essential job and not very stable."*

With a high proportion of the cohort experiencing job loss or unemployment at some stage throughout the pandemic, many participants were forced to look for new opportunities outside of their previous employment sector. For some qualitative interview participants, the impacts of COVID-19 on job security and income made them reassess their career choice. One participant described how the pandemic made it difficult to find a job in marketing and that this prompted her to reflect on her career direction more broadly and seek out new education and training opportunities.

Another participant, who worked in the travel industry, was made redundant due to COVID-19; and job security was a key motivating factor in changing his career. Similarly, another participant who also worked in the travel industry spoke about the employment uncertainty COVID-19 created when it came to returning to work after maternity leave, and hoped that "there's still a job when I'm done." Several participants spoke about the challenges of completing placements for degrees or finding a job especially for entry-level positions.

> *"…it was very hard to even just get interviews…I think no one was leaving their jobs, so there was no new jobs. … And then the ones that were a job that we had essentially 2 years of graduates trying to get them instead of one year, so it was this huge build-up of people just trying to get like one job."*

FINANCIAL HARDSHIP

One in three participants reported experiencing some form of financial hardship since March 2020. Of people who experienced financial hardship, at least 40% of participants required financial support from family and friends, 31% were unable to pay their utility bill and 31% received assistance from welfare or community organisations. Over time, there has been a downward trend of participants requiring financial help from friends or family. However, the proportion of people unable to pay their utilities bill increased between November 2020 and April 2021. Alarmingly, a small proportion of participants (2-5%) reported going without meals.

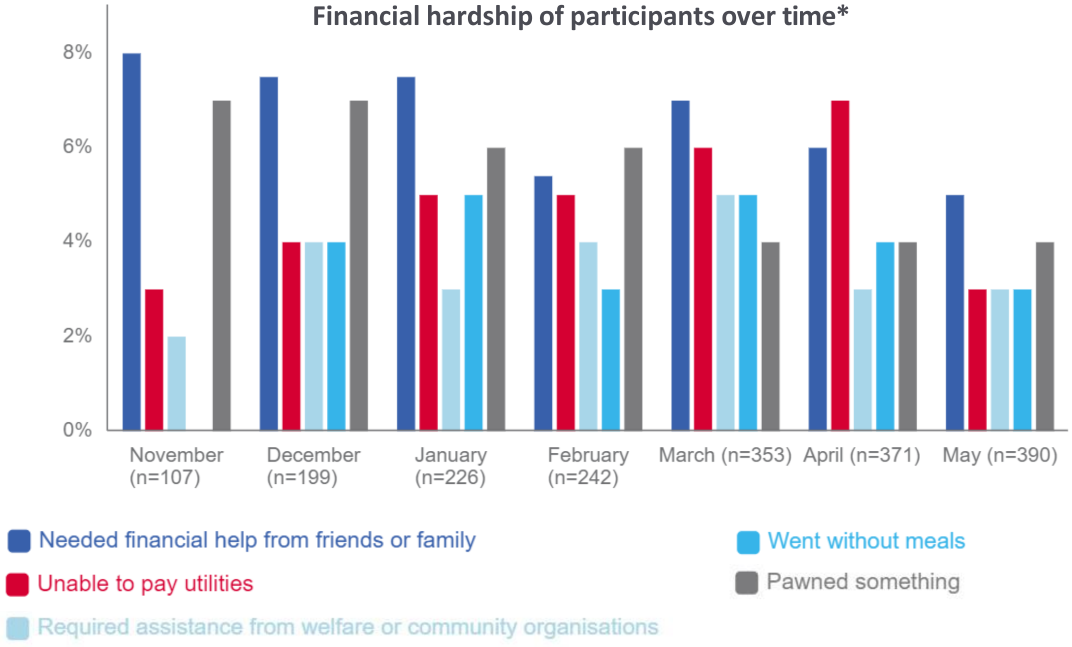

*Participants could select more than one form of financial hardship.

Some qualitative interview participants spoke about the support they provided to others, received from others, or received from the government during times of financial hardship in the pandemic. Participants highlighted the importance of family and friends for both financial and non-financial support.

The representative for international students within the Community Engagement Group reported that many international students were experiencing financial hardship because they had lost financial support from their families, and their work hours were restricted due to student visa requirements. Foodbanks and universities provided much-needed assistance for them. Other temporary visa holders were also at greater risk as they had no access to government benefits. Unfortunately, most participants from the Community Engagement Group did not feel secure about their income and finances.

"… I registered for free meal packs from the university and got groceries from foodbanks. I am not sure how I would survive without them." - International student

However, the Community Engagement Group also reported that there were groups who were less at risk of negative financial impacts due to the pandemic, such as retirees who had planned well for retirement, those who work in industries that have been positively affected by COVID-19 such as rural real estate, and people with access to family support.

ACCESS TO GOVERNMENT FINANCIAL SUPPORT

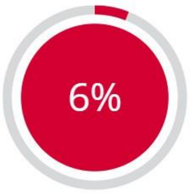

6% of participants reported accessing government financial support.

Six percent of participants reported that they had accessed government financial support. Women were more likely to report accessing financial support than men (7% and 3% respectively). A higher proportion of young people aged 18-25 accessed financial support than any other age group.

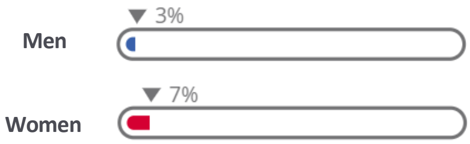

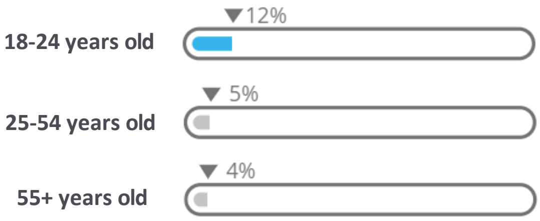

The qualitative interview participants noted the importance of government income support payments after losing work. However, one participant spoke about the uncertainty of government financial support at the start of the pandemic and ongoing concerns the support would be removed or reduced with limited warning. In addition, another participant mentioned the need to persevere when applying for income support payments after her daughter was initially deemed ineligible.

"It kind of felt like every press conference was just kind of waiting to see, 'Oh, are they going to drop the rate? Are they going to remove the eligibility?' …it felt like you were very much beholden to someone else's idea of how much support someone who's lost their job because of coronavirus would be…" - University student

"We just group together with our neighbours and do random Google searches." – School student

Participants from the Community Engagement Group remarked that information about the availability of different financial or practical supports could be hard to find or understand, and some of them had to rely on internet searches.

Other hurdles and deterrents to accessing financial support included difficulties determining eligibility, strict conditions for accessing payments, fear of judgment and difficulty predicting exact changes of annual income due to COVID-19. In addition, participants reported gaps in eligibility for financial support for international students, people who are homeless, and nursing students who acquired COVID-19 while on placement (and were thus ineligible for Work Cover payments).

"There's these broad-brush strokes of trying to support people but there's some real key gaps of where people are missing out. And they're the people with the smallest voice or the least ability of coming together to highlight what's going on" - Healthcare worker

COVID-19 WORK AND HEALTH STUDY

The COVID-19 Work and Health Study monitored the health and work of 2603 working-age Australians during 2020 and into 2021 [1]. The national cohort consists of a control group of individuals whose working hours were unaffected during the early stages of the pandemic, and a group of individuals that experienced work loss. All respondents were engaged in paid work prior to the pandemic. This section reports on the levels of financial stress experienced by a subset of 1006 respondents that completed questions on finances at four survey time-points.

FINANCIAL STRESS

During the early stages of the pandemic, the newly unemployed reported the highest levels of financial stress, followed by individuals that retained an employment relationship but were not currently working (i.e. temporarily stood down from work). To assess the level of financial stress, participants were asked the following question "What do you feel is the level of your financial stress today, on a scale of 1 to 10 where 1 is not at all stressed and 10 is as stressed as can be?" [2]. The results are reflected in the figure below.

Financial stress decreased over time, and stalled during second COVID-19 wave (i.e. Survey 3) for people who were out of work during the early stages of the pandemic. This decrease, over a 6-month period, was most marked for individuals that with the highest initial financial stress scores, namely those who were stood down from work or unemployed, compared to people that were working at Survey 1. Part of this may be due to people returning to work as restrictions eased. However, our findings show that significant gaps in levels of financial stress remain between the groups after 6 months, indicating ongoing stress among people who may be in less stable work. Previously, we have noted that access to financial resources† [2], reduce some of the negative mental health consequences upon pandemic-related work loss [1].

Financial stress of participants over time

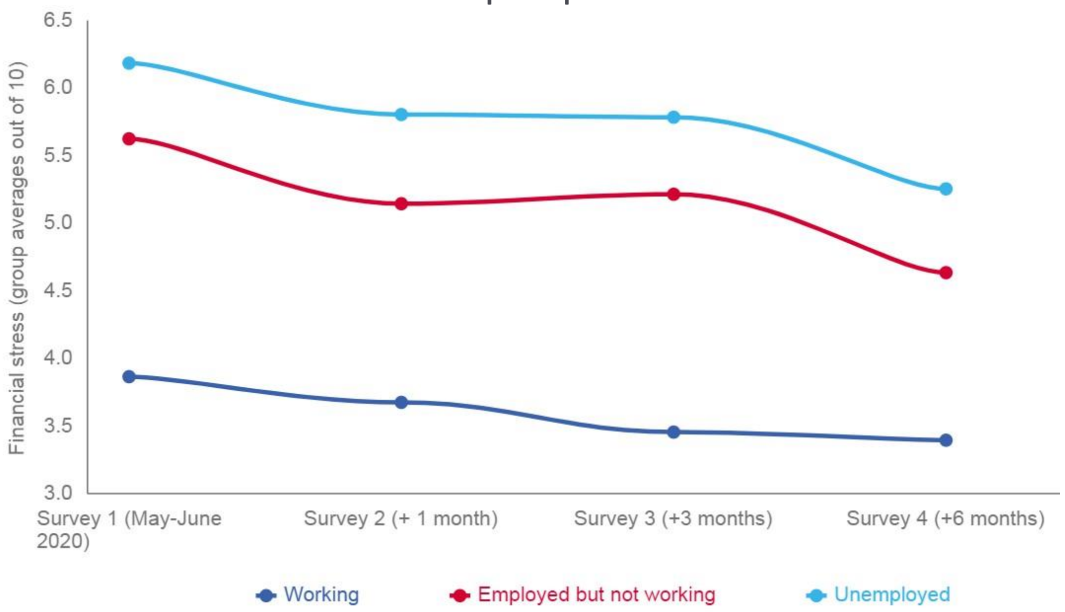

Individuals working at the time of Survey 1 also experienced a decrease in financial stress over the six-month period. This may represent an increase in disposable income from reduced consumer spending and also forms of savings from increased working from home arrangements. Correspondingly, workers experiencing financial stress were more likely to report concerns about losing the ability to work from home [3].

† *'If all of a sudden you had to get $2000 for something important, could the money be obtained within a week?'*

[1] Griffiths D, Sheehan L, van Vreden C, Petrie D, Grant G, Whiteford P, Sim MR, Collie A. The impact of work loss on mental and physical health during the COVID-19 pandemic: baseline findings from a prospective cohort study. Journal of occupational rehabilitation. 2021 Mar 3:1-8.
[2] Australian Bureau of Statistics. General Social Survey (GSS): Household Survey Questionnaire. Canberra: Australian Bureau of Statistics; 2014.
[3] Griffiths D, Sheehan L, van Vreden C, Whiteford P, Collie A. Returning to the workplace during the COVID-19 pandemic: The concerns of Australian workers. Journal of occupational rehabilitation. 2021 Jun 15:1-0.

SUMMARY

- COVID-19 has had a significant impact on income and finances for many people in our study.
- In May 2021, the majority of our cohort were employed in some capacity or had retired, while 8% were unemployed.
- Young people (aged 18-25 years) were more likely to be unemployed than any other age group.
- Many participants have had a change of income or work since the start of the pandemic.
- One in three participants reported experiencing some form of financial hardship since March 2020.
- Of those experiencing financial hardship at least 40% required financial support from family or friends.
- Very few of our participants accessed government income support.
- Participants noted challenges finding information about income support, understanding eligibility criteria or applying for payments.
- There have also been 'silver linings' with some participants reporting improvements in their work-life balance while others, such as essential workers, expressing gratitude at their ongoing ability to work and earn an income.

RECOMMENDATIONS

1. **Ensure that information about COVID-19 financial support is easy to understand and access.**
Participants from the Community Engagement Group suggested that information about COVID-19 financial support, including clear eligibility criteria, should be presented more clearly, in language that is easy to understand.

2. **Increase timely access to financial support by simplifying and streamlining procedures for accessing different support packages.**
Participants from the qualitative interviews and Community Engagement Group suggested suggested that financial support criteria and procedures to apply should also be simplified to ensure people who are eligible can access the support they need. As well, specific groups who are deemed ineligible for government support packages (ie. international students, refuges/asylum seekers, temporary visa holders, people who are homeless) should be linked to community organisations, foodbanks and universities, who can provide provided much-needed assistance for them.

REPORT PREPARED BY

Ms Freya Saich
Mr Edwin Kwong
Dr Katherine Heath
Ms Aimée Altermatt
Dr Stephanie Munari
Dr Bronwen Merner
Assoc Professor Sophie Hill
Dr Daniel Griffiths
Prof Alex Collie
Professor Mark Stoové
Dr Alisa Pedrana
Dr Katherine Gibney
Professor Margaret Hellard

ACKNOWLEDGEMENTS

Participants of the Community Engagement Group
Participants of the Qualitative Interviews
Optimise Data Collectors
Optimise Data Management Team
Optimise Qualitative Working Group
Optimise Knowledge Translation and Policy Working Group
Optimise Executive Committee

Burnet Institute
85 Commercial Road
Melbourne, Australia, 3004

burnet.edu.au

The Peter Doherty Institute
for Infection and Immunity
792 Elizabeth Street
Melbourne, Australia, 3000

doherty.edu.au

Chief Investigators

Professor Margaret Hellard AM
margaret.hellard@burnet.edu.au
+61 3 9282 2111

Dr Katherine Gibney
katherine.gibney@unimelb.edu.au
(03) 9035 3958

For More Information

Stephanie Fletcher-Lartey
Study Coordinator
stephanie.fletcher@burnet.edu.au
