# Supplementary material for: What Lies Beneath? The Role of Community Engagement in Translating COVID-19 Research Findings to Policy-Makers
Source: Int J Health Policy Manag. 2024 Apr 20;13:8249. doi: 10.34172/ijhpm.2024.8249 (PMC11270602; doi:10.34172/ijhpm.2024.8249)
Supplement: Supplementary file 3 — Example of CEG Meeting Summary. [file ijhpm-13-8249-s003.pdf]

Article title: What Lies Beneath? The Role of Community Engagement in Translating COVID-19 Research Findings to Policy-Makers

Journal name: International Journal of Health Policy and Management (IJHPM)

Authors' information: Bronwen Merner[1]*, Sophie Hill[1], Freya Saich[2], Ariane Virgona[1], Defeng Jin[2], Alisa Pedrana[3,4], Coral Keren[5], Rachel Kar Yee Chung[5], Deborah Osborne[2], Anna Lee Wilkinson[3,4,6], Alison Coelho[7], Lisa Gibbs[6,8], Katherine B. Gibney[9,10], Margaret Hellard[3,4,11,12], Dean Lusher[13], Rebecca Ryan[1]

[1]Centre for Health Communication and Participation, School of Psychology and Public Health, La Trobe University, Melbourne, VIC, Australia.

[2]Burnet Institute, Melbourne, VIC, Australia.

[3]Disease Elimination, Burnet Institute, Melbourne, VIC, Australia.

[4]School of Public Health and Preventive Medicine, Monash University, Melbourne, VIC, Australia.

[5]Community Engagement Group, Optimise Study, Burnet Institute, Melbourne, VIC, Australia.

[6]Melbourne School of Population and Global Health, University of Melbourne, Melbourne, VIC, Australia.

[7]Coelho Networks, Melbourne, VIC, Australia.

[8]Centre for Disaster Management and Public Safety, University of Melbourne, Melbourne, VIC, Australia.

[9]Victorian Infectious Diseases Service, Royal Melbourne Hospital, Melbourne, VIC, Australia.

[10]Department of Infectious Diseases, University of Melbourne, Peter Doherty
Institute for Infection and Immunity, Melbourne, VIC, Australia.

[11]Department of Infectious Diseases, The Alfred and Monash University, Melbourne, VIC, Australia.

[12]Doherty Institute and Melbourne School of Population and Global Health, University of Melbourne, Melbourne, VIC, Australia.

[13]Centre for Transformative Innovation, Swinburne University of Technology, Melbourne, VIC, Australia.

***Correspondence to:** Bronwen Merner; Email: b.merner@latrobe.edu.au

Citation: Merner B, Hill S, Saich F, et al. What lies beneath? The role of community engagement in translating COVID-19 research findings to policy-makers.Int J Health Policy Manag.2024;13:8249. doi:10.34172/ijhpm.2024.8249

Supplementary file 3. Optimise Study: Community Engagement Group Meeting

Optimise Study: Community Engagement Group Meeting

13th June 2021, 5-6.30pm

Topic: Income and finances

All participants agreed that the data about income and finances as presented in the Optimise report aligned with their expectations.

Impacts of COVID-19 on income and finances

Participants reported the groups more likely to have experienced negative impacts on their income and finances were:

- International students: "I am an international student and financially supported by my family. My family was not doing too great due to the pandemic and lockdown. I had to save money to pay rent and utility bills, so I registered for free meal packs from the university and got groceries from foodbanks. I am not sure how I would survive without them." Some students moved away from the city to suburbs where rent was cheaper. Some went home and are now studying online. It was difficult not being able to work more hours than allowed on the student visa, particularly when financial support from family is no longer available
- Other visa holders: this group were more likely to lose their jobs first, and also had no access to government benefits.
- People already experiencing significant disadvantage e.g. people who are homeless, people on JobSeeker. For these groups, finding a job became even harder during COVID, particularly during lockdowns. Foodbanks were useful when they could be accessed.
- Domestic students: many lost their part-time jobs when COVID first happened
- People in highly exposed jobs e.g. entertainment industry ("I went from one hundred to zero" in terms of employment), international education, hospitality
- People with long COVID who were unable to return to work in their previous capacity could experience a decreased income over a prolonged period

Groups who were less at risk of negative impacts on income and finances:

- Retirees who had planned well for retirement: this group could have less expenses than usual e.g. driving less, GP appointments bulk billed via telehealth, and had less opportunities to spend discretionary income
- Working in industries that have been positively affected by COVID e.g. rural real estate
- People with access to family support e.g. "bank of mum and dad"
- Healthcare workers: there is currently more work than is manageable

Difficulties accessing financial support:

- Information about the different financial or practical supports available could be hard to find and understand. One participant who lives in crisis accommodation reported they were not given any help from their accommodation provider to navigate support options, including food banks, and therefore relied on internet searches: "We just group together with our neighbours

and do random google searches." The opening hours of their closest food bank were difficult to find. For some people, this lack of support was further evidence that the government did not care about supporting them.

- Participants needing financial support reported it was difficult to determine if they were eligible for payments: "… understanding if they're eligible for particular payments, people aren't sure. A lot of them question whether their circumstances are that bad."
- Some participants reported the strict conditions for accessing payments were a deterrent e.g. needing to use all of your savings/annual leave first meant it wasn't always worth applying for payments
- Needing to predict exact changes in annual income as a result of COVID could be difficult
- Fears of being judged could also be a barrier for people applying for payments.
- Participants reported there were gaps in eligibility for financial support that meant people in need were being left out e.g. international students, people already experiencing significant disadvantage such as homelessness, nursing students who acquired COVID while on placement: "There's these broad brush strokes of trying to support people but there's some real key gaps of where people are missing out. And they're the people with the smallest voice or the least ability of coming together to highlight what's going on"
- Information about how to apply for different financial support was also difficult to understand: "I've tried to look at some of the websites, and I can't understand anything that I'm looking at".

Feelings of security regarding income and finances

- Most participants did not feel secure about their income and finances
- One participant suggested safer quarantine facilities and processes may help stimulate some sectors of the economy, such as international education, through facilitating the arrival of higher numbers of international students and other visitors
- The participant representing health care workers reported that although there was an excess of work in health care, workers were at-risk of burnout given the stress of COVID-19 and being unable to plan for a break (due to lockdowns, border restrictions etc). This may lead to some reducing their regular hours to cope.
- The participant representing international students welcomed the Federal Government's waiver of the 40-hour work limit for student visa holders working in essential sectors. However, she reported that a lot of employers were seeking permanent residents or Australian citizens only.
- The participant representing older people reported that people who have planned for their retirement may be managing well because their expenses are lower than anticipated. However, retirees who have lost their partner and are living alone may be at higher risk of emotional issues

Specific recommendations for improving COVID-related communication

- Simplify guidance for seeking support payments (including eligibility criteria)
- Highlighting the ongoing impacts of long COVID in vaccine advertising may help to persuade people to have the vaccination.
